# 2547. Targeting penicillin-binding protein 2 (PBP2) to treat metallo-beta-lactamase (MBL)-producing *Klebsiella pneumoniae*: Mecillinam as an American Ninja Warrior

**DOI:** 10.1093/ofid/ofad500.2164

**Published:** 2023-11-27

**Authors:** Jack F Klem, Pharm D Candidate, Jan Naseer Kaur, Nicholas M Smith, Al Chen, Patricia N Holden, Jürgen B Bulitta, Brian T Tsuji

**Affiliations:** University at Buffalo, Buffalo, New York; University at Buffalo, Buffalo, New York; University at Buffalo, School of Pharmacy & Pharmaceutical Sciences, Buffalo, New York; University at Buffalo, Buffalo, New York; University at Buffalo, School of Pharmacy & Pharmaceutical Sciences, Buffalo, New York; University of Florida, Gainesville, Florida; University at Buffalo, School of Pharmacy & Pharmaceutical Sciences, Buffalo, New York

## Abstract

**Background:**

‘Nightmare’ MBL gram-negatives resistant to nearly all antibiotics have emerged in the clinical setting and are a cause for concern. Here, we assessed the role of targeted PBP2 binding using mecillinam (MEC), which is currently available in other countries and has high affinity for PBP2, as an adjuvant to established β-lactam regimens to combat pan drug resistance.

**Methods:**

*Klebsiella pneumoniae* harboring *bla*_NDM-1_, *bla*_CMY-6_, *bla*_CTX-M-15_, and *bla*_SHV-2_ was exposed to an array of therapeutic drug regimens in a Hollow Fiber Infection Model (HFIM) at a starting inoculum of ∼10^7^ CFU/mL over 168h. These arms included MEC monotherapy (2hPI Q8H, target C_max,ss_ = 32 ug/mL) in addition to combination regimens that contained aztreonam (ATM) (2g 2hPI Q8H) + ceftazidime/avibactam (CAZ/AVI) (2g/0.5g 2hPI Q8H) ± MEC (2hPI Q8H, target C_max,ss_ = 32 ug/mL). All drugs were discontinued at 168h, after which populations were assessed for regrowth for 48h. Samples were also collected, fixed, and imaged under a scanning electron microscope (SEM, Carl Zeiss Auriga).

**Results:**

The MEC monotherapy arm regrew to carrying capacity (∼10^10^ CFU/mL) in 24h whereas combination arms demonstrated bactericidal activity throughout the duration of drug treatment. While 24h killing rates were comparable between the two combination arms, 168h counts were ∼3.0 log_10_ CFU/mL lower in ATM + CAZ/AVI + MEC than in ATM + CAZ/AVI (10^2.90^ CFU/mL vs. 10^5.92^ CFU/mL, respectively). Both combination arms recovered following drug discontinuation; however, this recovery was delayed in ATM + CAZ/AVI + MEC as was supported by a ∼6.5 log_10_ CFU/mL difference in 192h counts (10^9.51^ CFU/mL in ATM + CAZ/AVI vs. 10^2.98^ CFU/mL in ATM + CAZ/AVI + MEC). 192h SEMs supported this observation as rods predominated in the ATM + CAZ/AVI sample whereas more debris/fewer cells were apparent in the ATM + CAZ/AVI + MEC sample. Spheroplasts were observed throughout drug treatment in all samples treated with MEC.

**Conclusion:**

The targeted PBP2 binding capacity of MEC may be a promising adjuvant to β-lactam/β-lactamase inhibitor combinations. Non-traditional β-lactam strategies that enhance the existing antibiotics may be of paramount importance for clinicians to combat MBL gram negative pan drug resistance.

**Disclosures:**

**All Authors**: No reported disclosures

